# Amphetamine Promotes Cortical Up State in Part Via Dopamine Receptors

**DOI:** 10.3389/fphar.2021.728729

**Published:** 2021-08-19

**Authors:** Guofang Shen, Wei-Xing Shi

**Affiliations:** ^1^ Department of Pharmaceutical and Administrative Sciences, Loma Linda University School of Pharmacy, Loma Linda, CA, United States; ^2^ Department of Basic Sciences, Loma Linda University School of Medicine, Loma Linda, CA, United States

**Keywords:** amphetamine, prefrontal cortex, Up state, slow oscillation, dopamine, norepinepherine, cortical state, attention

## Abstract

Cortical neurons oscillate between Up and Down states during slow wave sleep and general anesthesia. Recent studies show that Up/Down oscillations also occur during quiet wakefulness. Arousal eliminates Down states and transforms Up/Down oscillations to a persistent Up state. Further evidence suggests that Up/Down oscillations are crucial to memory consolidation, whereas their transition to a persistent Up state is essential for arousal and attention. We have shown that D-amphetamine promotes cortical Up state, and the effect depends on activation of central α_1A_ adrenergic receptors. Here, we report that dopamine also plays a role in D-amphetamine’s effect. Thus, using local-field-potential recording in the prefrontal cortex in chloral hydrate-anesthetized rats, we showed that the Up-state promoting effect of D-amphetamine was attenuated by antagonists at either D1 or D2-like dopamine receptors. The effect was also partially mimicked by co-activation of D1 and D2-like receptors. These results are consistent with the fact that D-amphetamine increases the release of both norepinephrine and dopamine. They are also in agreement with studies showing that dopamine promotes wakefulness and mediates D-amphetamine-induced emergence from general anesthesia. The effect of D-amphetamine was not mimicked, however, by activation of either D1 or D2-like receptors alone, indicating an interdependence between D1 and D2-like receptors. The dopamine/norepinephrine precursor L-DOPA also failed to promote the Up state. While more studies are needed to understand the difference between L-DOPA and D-amphetamine, our finding may provide an explanation for why L-DOPA lacks significant psychostimulant properties and is ineffective in treating attention-deficit/hyperactivity disorder.

## Introduction

During slow wave sleep and general anesthesia, the membrane potential of cortical neurons alternates between two preferred levels known as the Up and Down states (Neske, 2016; Poulet and Crochet, 2019). During the Up state, cortical neurons are depolarized and bombarded with synaptic inputs, whereas during the Down state, they are hyperpolarized and synaptically quiescent. In an electroencephalogram (EEG), the transitions between Up and Down states are reflected as large-amplitude, low-frequency oscillations known as slow oscillations.

Up/Down or slow oscillations also occur during quiet wakefulness, though they are brief in duration and localized in discrete areas (Petersen et al., 2003; Poulet and Petersen, 2008; Vyazovskiy et al., 2011; Polack et al., 2013; Einstein et al., 2017). Their occurrence increases with increasing sleep pressure (Nir et al., 2011; Vyazovskiy et al., 2011). Arousal or locomotion eliminates the Down state, leading to a persistent depolarization (see review by Poulet and Crochet, 2019). A transition from Up/Down oscillations to a persistent Up state also occurs during the transition from sleep to wake or from general anesthesia to an arousal state (Steriade et al., 2001; Constantinople and Bruno, 2011). Since the awake Up/Down oscillations are local, they are not readily detectable by a regular scalp EEG. Consequently, the awake EEG is dominated by low-amplitude, high-frequency activities associated with the Up state.

Evidence suggests that Up/Down oscillations during sleep and quiet wakefulness are crucial to memory consolidation, whereas their transition to a persistent Up state is essential for arousal and attention (Harris and Thiele, 2011; Lee and Dan, 2012; Mcginley et al., 2015). In chloral hydrate-anesthetized rats, we have shown that D-amphetamine as well as methylphenidate dose-dependently increases the time that the prefrontal cortex (PFC) spends in the Up state (T_UP_) (Shen and Shi, 2021). Our evidence further suggests that this Up-state promoting effect of D-amphetamine depends on activation of central α_1A_ adrenergic receptors. However, the selective dopamine (DA) transporter blocker GBR12909 also increases T_UP_, though its effect is smaller than that induced by a selective norepinephrine (NE) reuptake inhibitor (Shen and Shi, 2021). This finding and the fact that D-amphetamine increases the release of both NE and DA (Kuczenski and Segal, 1997; Rothman et al., 2001) point to the possibility that D-amphetamine promotes cortical Up state via both NE and DA receptors.

In this study, we tested the above possibility by administering selective DA receptor antagonists either before or after D-amphetamine injection. We also tested whether the effect of D-amphetamine is mimicked by activation of D1 and D2-like receptors using selective DA receptor agonists and the DA/NE precursor L-DOPA. Our results showed that blockade of either D1 or D2-like receptors attenuated the effect of D-amphetamine, whereas co-activation of D1 and D2-like receptors partially mimicked the effect. Unexpectedly, L-DOPA decreased T_UP_, instead of increasing it. These results provide new insight into the mechanism of action of D-amphetamine and may have important clinical implications.

## Materials and Methods

All experimental procedures were approved by Animal Care and Use Committee of Loma Linda University and performed in accordance with the Guide for the Care and Use of Laboratory Animals as adopted and promulgated by the National Institutes of Health. Male Sprague-Dawley rats weighing 250–400 g (2.5–5 months old, Envigo, Indianapolis, IN) were anesthetized with chloral hydrate (400 mg/kg, i. p., followed by 15 mg/kg, i. v., every 10–20 min). Throughout the experiment, body temperature was maintained at 36–37°C with a homeothermic blanket system (Harvard Apparatus, Holliston, MA). Local field potentials (LFPs) were recorded using methods described previously (Dela Peña et al., 2018; Shen et al., 2018; Shen and Shi, 2021). Briefly, glass electrodes were filled with sodium acetate (0.5 M) and pontamine sky blue (1%) and lowered into the medial PFC (from bregma: AP 2.8–3.2 mm, ML 0.5–1 mm, DV 2.5–3.5 mm) using a motorized microdrive (864/1, Harvard Apparatus). Signals were amplified (A-M System Model 3000, Sequim, WA), bandpass-filtered (0.1–200 Hz), digitized at 20 kHz (Digidata 1550, Molecular Devices, Sunnyvale, CA), and stored to a computer using Clampex 10.4 (Molecular Devices). At the end of recording, the recording site was marked by iontophoretic ejection of pontamine sky blue (−20 μA for 10–15 min) and verified using standard histological method. All drugs were purchased from Sigma-Aldrich (St. Louis, MO) and administered through a lateral tail vein. Stock drug solutions were prepared using water or 25% polyethylene glycol 200 (prazosin), stored in a −80° freezer, and diluted with saline immediately before injection.

Data analyses were performed using IBM SPSS Statistics and in-house programs written in Python and Visual Basic for Applications in Microsoft Excel. The sign of LFPs was reversed so that upward deflections represent transitions to Up states. The slow drift in LFPs, caused by a 0.1 Hz high-pass filter used during recording, was estimated and removed using the Asymmetric Least Squares Smoothing method (Shen et al., 2018; Shen and Shi, 2021). The voltage distribution of LFPs was computed every 10 s. LFPs above 0 mV were considered in Up states. To construct the spectrogram, recordings were divided into 10.24 s long segments with 50% overlap. After tapering using the Hanning window function and removal of the linear trend, the Fast Fourier Transform was performed on each segment to yield autospectra with a resolution of 0.098 Hz. The intensity of Up/Down or slow oscillations was measured by the absolute mean power (mV^2^) between 0.3 and 1.5 Hz (P_SO_).

All numerical results were based on analysis of 2 min recordings selected during baseline and after each drug injection. Effects of drugs were assessed using the linear mixed models (MIXED) in which intercepts of individual animals were treated as the random effect and drug doses as a categorical or continuous variable. When doses were treated as a continuous variable, they were log-transformed to achieve linearity of the dose-response curve. Models were selected based on the Akaike’s Information Criterion. Differences between doses were determined by comparing the marginal means using Bonferroni post hoc tests. The marginal means were estimated after fitting the data to a MIXED model. To meet the assumption of residual normality and homoscedasticity, data were sometimes transformed using a parametric (Box-Cox, Yeo-Johnson, or logit transformation) or nonparametric (quartile transformation) method. The latter was used only when all three parametric methods failed. Numerical results are expressed as the mean ± the standard error of the mean. A *p* value less than 0.05 is considered statistically significant.

## Results

### Dependence of D-Amphetamine’s Effect on Baseline State

**FIGURE 1:**
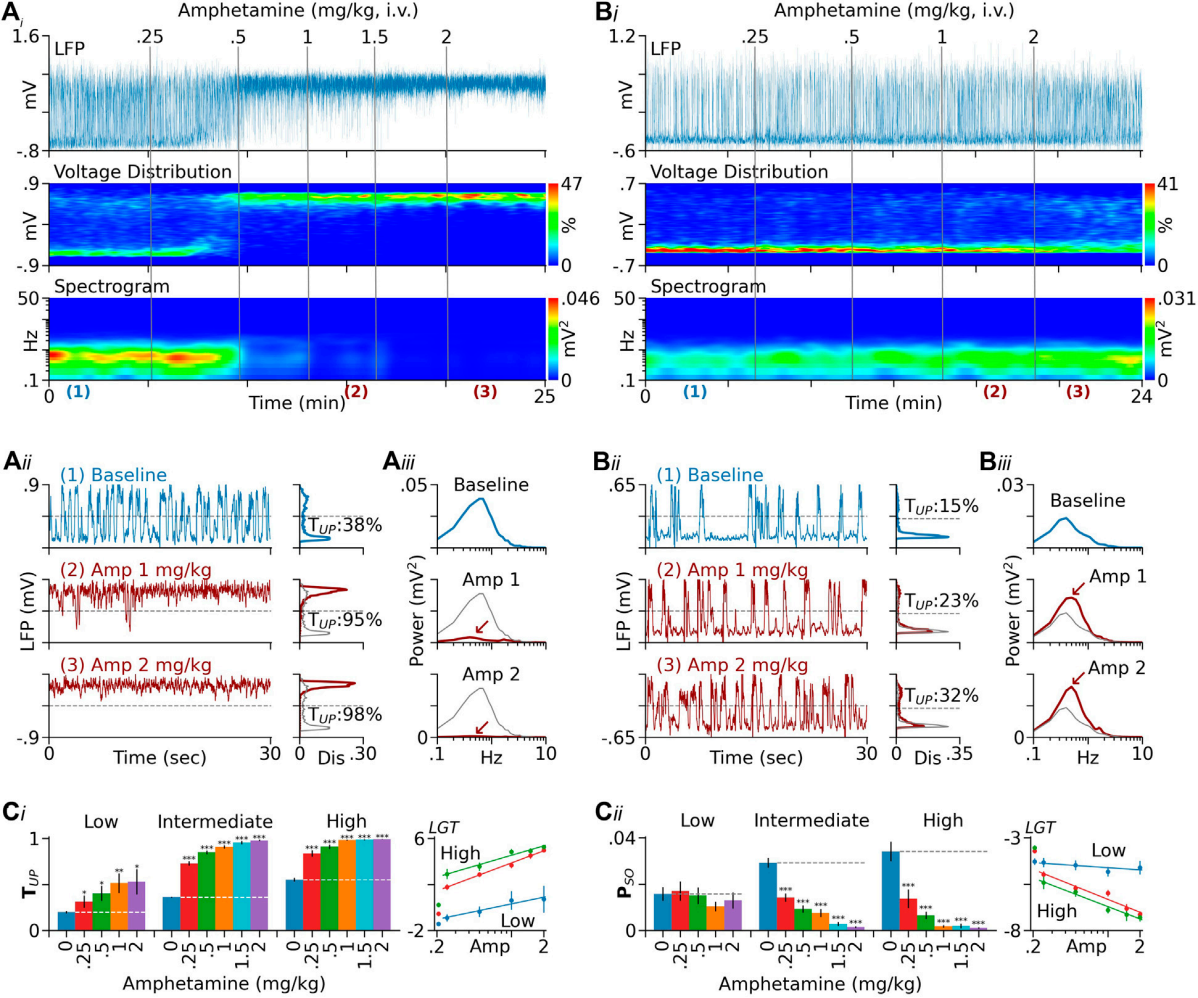
Dependence of D-amphetamine’s effect on baseline T_UP_. **(Ai)** Results from a rat showing changes induced by D-amphetamine in PFC LFPs (top), LFP voltage distribution (middle), and spectrogram (bottom). In this and following figures, the sign of LFPs is reversed so that upward deflections represent transitions to Up states. The vertical lines indicate the times of drug injection. The numbers above the lines are cumulative doses (mg/kg). **(Aii)** Left traces are segments of LFPs displayed on an expanded timescale. Charts on the right are distribution histograms of 2-min LFP recordings selected during baseline and after D-amphetamine injection. The locations of the selected recordings are indicated by the numbers below the spectrogram in **(Ai)**. For comparison, the baseline histogram is also shown in grey in histograms obtained after D-amphetamine. In this rat, D-amphetamine increased T_UP_ (the percentage of time in Up states) from 38 to 95% and then to 98% after 1 and 2 mg/kg, respectively. **(Aiii)** Autospectra of the same 2-min LFP recordings. The major spectral peak seen during baseline (upper chart) confirms the presence of Up/Down oscillations. The peak was almost completely suppressed after 2 mg/kg of D-amphetamine (red arrow). **(Bi–Biii)** Results from another rat showing that D-amphetamine was less effective in promoting the Up state in this rat compared to Rat A. This rat also had a low baseline T_UP_ (15%) relative to that of Rat A (38%). **(Ci)** Summary of data from 126 rats tested with D-amphetamine. They were divided into three groups based on baseline T_UP_ (low: <25%, intermediate: 25–45%, and high: >45%). While D-amphetamine increased T_UP_ in all groups (left), its dose-response curve is significantly downshifted in the low-baseline group (right). **(Cii)** D-Amphetamine had no significant effect on P_SO_ (the absolute mean power between 0.3 and 1.5 Hz) in the low-baseline group, but it significantly inhibited P_SO_ in both the intermediate and high-baseline groups (left). The dose-response curve of D-amphetamine is significantly upshifted in the low-baseline group relative to those in the intermediate and high-baseline groups (right, see Results for detailed statistics). In both **(Ci)** and **(Cii)**, the *y*-axis values in dose-response curves are logit-transformed (LGT). The lines are logarithmic fits to the means. The leftmost points are baseline values. *p < 0.05, **p < 0.01, ***p < 0.001 compared to baseline (blue bars).

Confirming our previous finding (Shen and Shi, 2021), D-amphetamine increased T_UP_ (the percentage of time that the PFC spent in the Up state) and decreased P_SO_ (the absolute mean power of slow oscillations between 0.3 and 1.5 Hz). In this study, we also found that the effect of D-amphetamine depended on baseline T_UP_, i.e., D-amphetamine was less effective in promoting the Up state in rats with a low baseline T_UP_ than in those with a high baseline T_UP_ (Figures 1A,B). To confirm this finding, we divided rats into three groups based on their baseline T_UP_ (<25%, *n* = 10, between 25 and 45%, *n* = 89, and >45%, *n* = 27). While d-amphetamine increased T_UP_ in all three groups (Figure 1Ci, right), its dose-response curve was significantly downshifted in the low-baseline group relative to those in the intermediate (intercept F_1,130_ = 90.2, *p* < 0.001, slope F_1,324_ = 18.37, *p* < 0.001) and high baseline groups (intercept F_1,67_ = 37.13, *p* < 0.001, slope F_1,95_ = 0.037, *p* = 0.847). The latter two groups also differed from each other in slope (F_1,282_ = 3.892, *p* < 0.05), but not in intercept (F_1,125_ = 3.467, *p* = 0.065). Similar results were obtained with P_SO_. Thus, D-amphetamine produced no significant effects on P_SO_ in the low-baseline group, but it significantly suppressed P_SO_ in both the intermediate and high-baseline groups (Figure 1Cii, left chart). The dose-response curve of D-amphetamine on P_SO_ was significantly upshifted in the low-baseline group compared to the intermediate (intercept F_1,108_ = 12.85, *p* < 0.001, slope F_1,224_ = 9.772, *p* < 0.01) and high-baseline groups (intercept F_1,43_ = 25.92, *p* < 0.001, slope F_1,96_ = 6.465, *p* < 0.05, Figure 1Cii). No significant differences were found between the intermediate and high-baseline groups (intercept F_1,124_ = 1.949, *p* = 0.165, slope F_1,279_ = 0.406, *p* = 0.524).

The dependence of D-amphetamine’s effect on baseline state may have important clinical implications (see Discussion). It also highlights the need to statistically control for baseline differences between animals. In this study, we included baseline T_UP_ as a covariate in all statistical analyses and compared D-amphetamine’s effect only between animals with similar baselines.

### Blockade of Dopamine Receptors Attenuated the Effect of D-Amphetamine

#### SCH23390 Pretreatment Attenuated the Effect of D-Amphetamine

**FIGURE 2:**
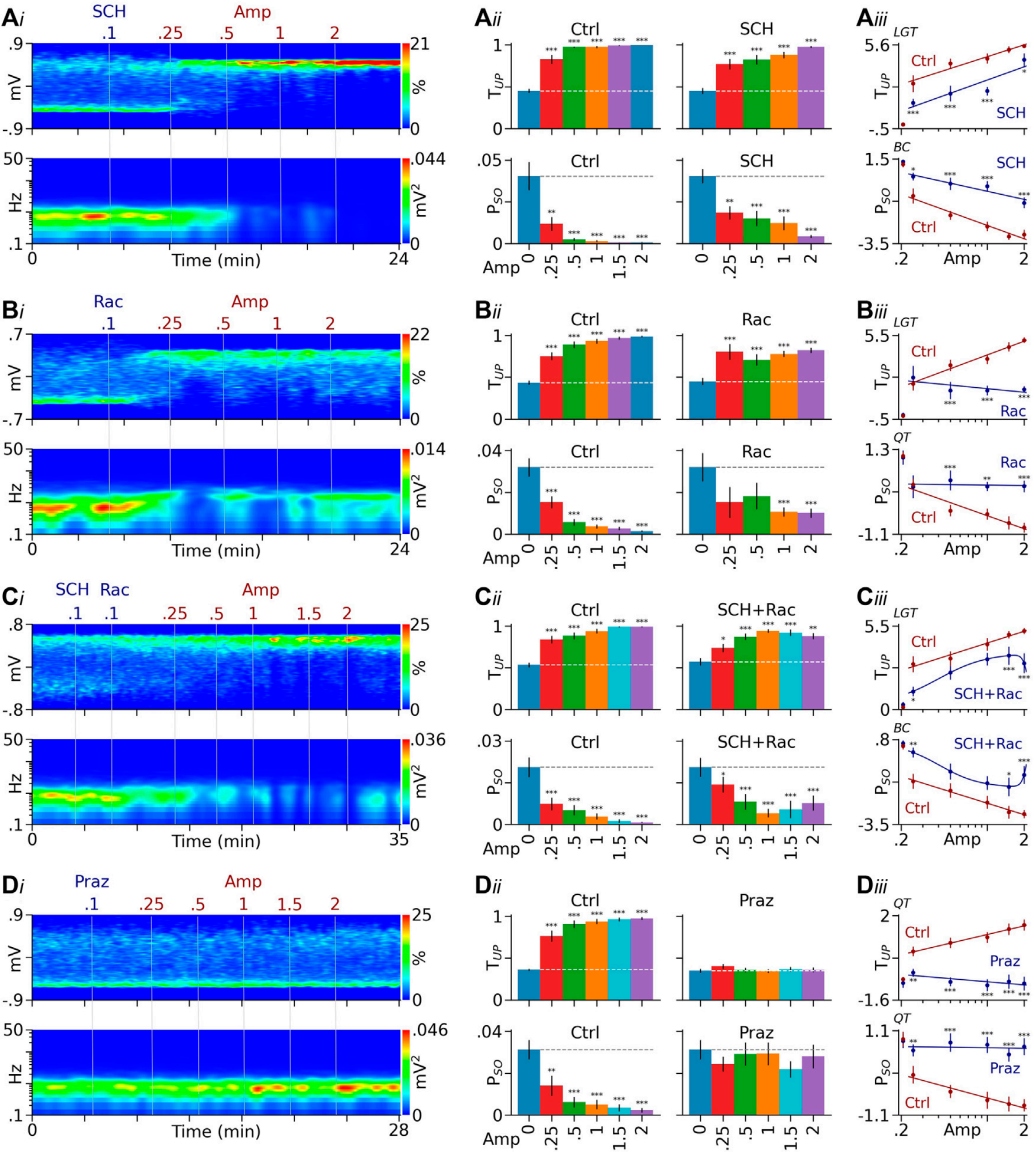
DA receptor antagonists attenuate D-amphetamine’s effects. **(Ai)** Heatmaps from a typical rat showing that after a pretreatment with the D1-like receptor antagonist SCH23390 (SCH), D-amphetamine (Amp) remained effective in promoting PFC Up state (top) and suppressing Up/Down oscillations (bottom). **(Aii)** Summary bar graphs showing that D-amphetamine’s effects on T_UP_ and P_SO_ were significant in both control (Ctrl) and SCH23390-pretreated rats (SCH). **(Aiii)** Compared to controls, however, SCH23390 pretreatment significantly reduced the effect of D-amphetamine (see Results for detailed statistics). **(Bi)** Results from a separate rat showing that the effect of D-amphetamine also persisted after blockade of D2-like receptors by raclopride (Rac). **(Bii, Biii)** Compared to controls, however, raclopride-pretreatment significantly reduced the effect induced by high doses of D-amphetamine. **(Ci)** Heatmaps from another rat showing that D-amphetamine’s effect continued after a pretreatment with both SCH23390 and raclopride. **(Cii, Ciii)** Compared to controls, the pretreatment reduced the effect induced by low and high, but not intermediate, doses of D-amphetamine. **(Di)** Data from another rat showing that the α1 receptor antagonist prazosin (Praz) completely prevented D-amphetamine from promoting the Up state and suppressing the slow oscillation. **(Dii, Diii)** Compared to controls, prazosin blocked the effect induced by all doses of D-amphetamine tested. The *y*-axis values in dose-response curves are logit (LGT), Box-Cox (BC), or quartile-transformed (QT). *p < 0.05, **p < 0.01, ***p < 0.001 vs baseline (blue bars) or the same dose of D-amphetamine in control rats (red dots).

To test whether D1-like receptors play a role in D-amphetamine’s effects, we pretreated rats with the D1-like receptor SCH23390 (0.1 mg/kg) and then injected D-amphetamine (0.25–2 mg/kg). SCH23390 by itself produced no significant effect on T_UP_ (from 45.3 ± 2.3% to 45.0 ± 3.4%, F_1,6_ = 1.161, *p* = 0.323) and P_SO_ (from 42 ± 5 to 40 ± 4mV^2^ × 10^−3^, F_1,6_ = 0.48, *p* = 0.514, *n* = 6, Figures 2Ai,ii). After SCH23390, D-amphetamine dose-dependently increased T_UP_ and decreased P_SO_ (Figures 2Ai,ii ). Compared to baseline-matched controls (*n* = 12), however, D-amphetamine’s effects were significantly reduced in SCH23390-pretreated rats (T_UP_: intercept F_1,20_ = 23.37, *p* < 0.001, slope F_4,81_ = 3.957, *p* < 0.01; P_SO_: intercept F_1,20_ = 27.61, *p* < 0.001, slope F_1,81_ = 80.65, *p* < 0.001, Figure 2Aiii).

#### Raclopride Pretreatment Also Attenuated the Effect of D-Amphetamine

To study the role of D2-like receptors, we pretreated a separated group of rats with the D2-like receptor antagonist raclopride (0.1–0.2 mg/kg) which, by itself, slightly increased T_UP_ (from 35.5 ± 1.7 to 47.8 ± 4.6%, F_1,11_ = 18.79, *p* < 0.001) and had no significant effect on P_SO_ (from 36 ± 6 to 32 ± 7 mV^2^×10^−3^, F_1,11_ = 0.788, *p* = 0.394, *n* = 10, Figure 2Bi). In raclopride-pretreated rats, D-amphetamine’s effects persisted (Figures 2Bi,ii), but they were significantly reduced compared to control animals (*n* = 20, T_UP_: intercept F_1,35_ = 19.07, *p* < 0.001, slope _1,129_ = 13.85, *p* < 0.001; P_SO_: intercept F_1,34_ = 9.275, *p* < 0.01, slope _1,129_ = 6.611, *p* < 0.001, Figure 2Biii). Pairwise comparisons suggest that raclopride preferentially attenuates the effects induced by high doses of D-amphetamine (Figure 2Biii).

To block both D1 and D2-like receptors, we treated another group of rats with both SCH23390 and raclopride (*n* = 9). The treatment slightly increased T_UP_ (from 44.6 ± 1.7 to 57.2 ± 4.5%, F_1,9_ = 13.41, *p* < 0.001) and decreased P_SO_ (from 26 ± 3 to 21 ± 3 mV^2^ × 10^−3^, F_1,9_ = 13.41, *p* < 0.001, Figure 2Ci). Subsequent injections of D-amphetamine promoted the Up state (Figures 2Ci,ii), but the effect was reduced compared to non-treated controls (Figure 2Ciii). Pairwise comparisons suggest that SCH23390/raclopride co-treatment reduces effects induced by low and high, but not intermediate, doses of D-amphetamine (Figure 2Ciii).

#### Prazosin Pretreatment Completely Blocked the Effect of D-Amphetamine

For comparison, we also examined the effect of the α1 receptor antagonist prazosin (0.1 mg/kg) which has been shown to block D-amphetamine’s effects on PFC Up state (Shen and Shi, 2021). Prazosin by itself slightly decreased T_UP_ (from 39.8 ± 2.0 to 35.1 ± 2.2%, F_1,7_ = 8.534, *p* < 0.05) and produced no significant effect on P_SO_ (F_1,7_ = 0.553, *p* = 0.482, *n* = 7, Figure 2Di). Confirming previous results, prazosin completely prevented D-amphetamine from increasing T_UP_ (F_5,17_ = 2.231, *p* = 0.097) and decreasing P_SO_ (F_5,8_ = 0.964, *p* = 0.492, Figures 2Dii,iii).

Taken together, the above results suggest that both D1 and D2-like receptors are involved in the Up-state promoting effect of D-amphetamine. However, unlike the α1 receptor antagonist prazosin which completely blocked the effect of D-amphetamine, DA receptor antagonists only attenuated the effect. The above results also suggest that DA’s contribution to D-amphetamine’s effect varies depending on the dose of D-amphetamine.

### Effects of Dopamine Receptor Antagonists Injected After Low and High Doses of D-Amphetamine

To directly examine the possibility that DA’s role varies depending on the dose of D-amphetamine, we injected SCH23390 or raclopride after low and high doses of D-amphetamine. Since effects of SCH23390 and raclopride were determined by comparing the activity before and after their injection in the same animals, this design reduced the variance caused by the differences in baseline between animals.

#### SCH23390 Was More Effective in Reversing the Effects Induced by Low Doses of D-Amphetamine Than Those Induced by High Doses

**FIGURE 3:**
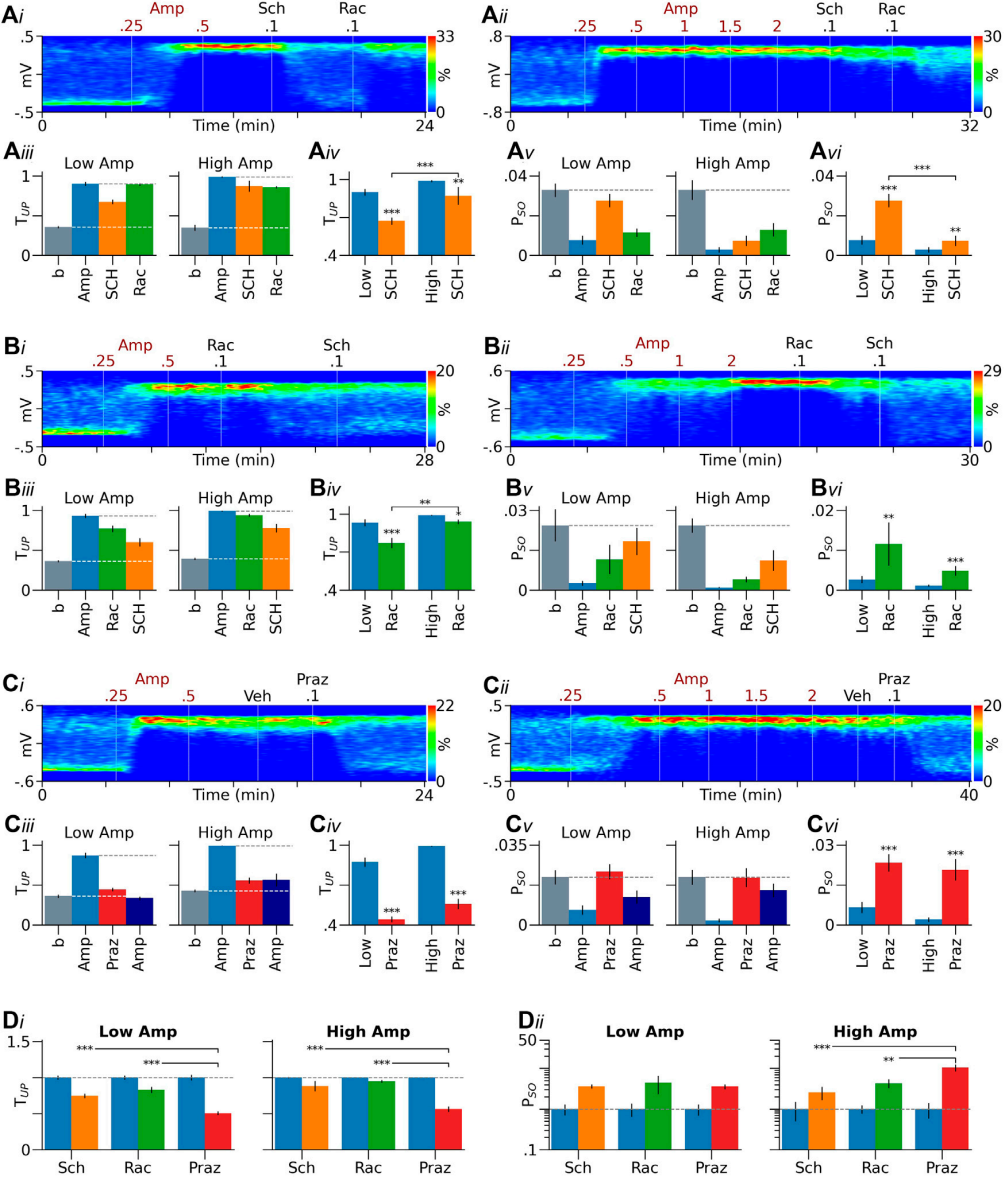
Role of DA receptors in effects induced by low and high doses of D-amphetamine. **(Ai–Aii)** Heatmaps from two representative rats showing that the D1-like receptor antagonist SCH23390 (SCH) more effectively reversed the effect induced by a low dose of D-amphetamine (Amp, A1) than that induced by a high dose **(Ai-ii)**. **(Aiii)** Summary bar graphs showing that in both low and high-amphetamine groups, SCH23390 partially reversed the increase in T_UP_ induced by D-amphetamine. The D2-like receptor antagonist raclopride (Rac), injected after SCH23390, increased T_UP_ in the low-amphetamine group and produced no further effects in the high-amphetamine group. **(Aiv)** MIXED analysis using values obtained after D-amphetamine as covariates (blue bars) suggests that SCH23390’s effect is significantly larger in the low-amphetamine group than in the high-amphetamine group. **(Av, Avi)** Similar results were obtained with P_SO_ (see Results for more detailed description). **(Bi–Bvi)** Raclopride was also more effective in reversing D-amphetamine’s effect in the low-amphetamine group than in the high-amphetamine group. SCH23390, given after raclopride, further reversed the effect of D-amphetamine. **(Ci–Cvi)** Prazosin (Praz), but not its vehicle (Veh), significantly reversed the effect of D-amphetamine. No significant differences were found in prazosin’s effect between the low and high-amphetamine groups. After prazosin, injections of additional doses of D-amphetamine (2.5–4.5 mg/kg) slightly decreased T_UP_ and P_SO_ in the low-amphetamine group and produced no further effects in the high-amphetamine group. **(Di, Dii)** In both the low and high-amphetamine groups, SCH23390 and raclopride were significantly less effective than prazosin in reversing D-amphetamine’s effect on T_UP_ **(Di)**. They were also less effective than prazosin in reversing D-amphetamine’s effect on P_SO_ in the high, but not low-amphetamine group **(Dii)**. Data are normalized by the means obtained after D-amphetamine injection (blue bars). *p < 0.05, **p < 0.01, ***p < 0.001 compared to D-amphetamine (blue bars in **Aiv, Avi, Biv, Bvi, Civ, Cvi**), the high amphetamine group **(Aiv, Avi, Biv)**, or prazosin **(Di, Dii)**.

Figures 3Ai,ii show results from two rats. In the first rat, SCH23390 was injected after a low dose of D-amphetamine (0.5 mg/kg), and it significantly reversed D-amphetamine’s Up-state promoting effect (Figure 3Ai). In the second rat, SCH23390 was injected after a high dose of D-amphetamine (2 mg/kg), and it produced only a limited effect (Figures 3Aii–vi). Figures 3Aiii-vi summarize the results from all rats tested with SCH23390. For simplicity, data obtained with 0.5 and 1 mg/kg of D-amphetamine were combined since they were statistically not different. In both the low (0.5 and 1 mg/kg) and high-amphetamine (2 mg/kg) groups, SCH23390 partially reversed the increase in T_UP_ induced by D-amphetamine (low: *p* < 0.001, *n* = 33; high: *p* < 0.01, *n* = 9). To determine the difference in SCH23390’s effect between the two groups, we compared T_UP_ measured after SCH23390 injection between the two groups using a univariate MIXED analysis in which T_UP_ measured immediately before SCH23390 injection was used as a covariate. The results suggest that SCH23390’s effect is significantly larger in the low-amphetamine group than in the high-amphetamine group (F_1,42_ = 14.85, *p* < 0.001, Figure 3Aiv). Unexpectedly, raclopride, given after SCH23390, reversed the effect of SCH23390 in the low (*p* < 0.001) but not high-amphetamine group (*p* = 1, Figure 3Aiii).

SCH23390 produced similar effects on P_SO_ (Figures 3Av–vi). Thus, it significantly reversed the decrease in P_SO_ induced by D-amphetamine. The effect was larger in the low-amphetamine group than in the high-amphetamine group (F_1,42_ = 23.79, *p* < 0.001, Figure 3Avi). Raclopride reversed the effect of SCH23390 in the low (*p* < 0.001) but not high-amphetamine group (*p* = 1, Figure A3v).

#### Raclopride Was Also More Effective in Reversing the Effects Induced by Low Doses of D-Amphetamine Than Those Induced by High Doses

In the above experiments, raclopride may reverse SCH23390’s effects by blocking D2-like receptors on DA neurons (DA autoreceptors). We have previously shown that raclopride, by blocking DA autoreceptors, not only prevents D-amphetamine from inhibiting DA neurons, but also enables D-amphetamine to excite DA neurons via α1 receptors (Shi et al., 2000a). The excitation may reverse the effect of SCH23390 by increasing DA release from DA neurons. To test this possibility, we injected raclopride immediately after D-amphetamine. We hypothesized that raclopride, by blocking DA autoreceptors, enhances D-amphetamine’s effect, particularly when it is injected after a low dose of D-amphetamine. We found, however, that raclopride partially reversed D-amphetamine’s effects in both the low and high-dose groups (low *p* < 0.001, *n* = 12; high *p* < 0.05, *n* = 12, Figures 3Bi–vi). Furthermore, the reversal was significantly larger in the low-amphetamine group than in the high-amphetamine group (F_1,24_ = 9.474, *p* < 0.01, Figures 3Biii–iv). SCH23390, given after raclopride, further decreased T_UP_ in both groups (low *p* < 0.05, high *p* < 0.01). Raclopride also partially reversed the effect of D-amphetamine on P_SO_ (low *p* < 0.01, high *p* < 0.001), but the effect was not significantly different between the low and high-amphetamine groups (F_1,24_ = 1.396, *p* = 0.249, Figure 3vi). In both groups, SCH23390, injected after raclopride, produced no significant further effects on P_SO_ (low *p* = 0.1, high *p* = 0.11).

The above results support the suggestion that both D1 and D2-like receptors are involved in D-amphetamine’s effects, but they are inconsistent with the above autoreceptor hypothesis, suggesting that D2-like receptors on DA target neurons also play a critical role in the effect of D-amphetamine. These results are also inconsistent with the pretreatment experiment shown in Figure 2B which suggests that raclopride preferentially reduces effects induced by high, but not low, doses of D-amphetamine. This inconsistency raises the possibility that different processes are involved in the initiation and maintenance of D-amphetamine’s effects, and DA’s role varies depending on not only the dose of D-amphetamine, but also the stage of D-amphetamine’s effect.

#### Prazosin Was Equally Effective in Reversing the Effects Induced by Low and High Doses of D-Amphetamine

For comparison, we injected prazosin after D-amphetamine in separate groups of animals (Figures 3Ci–vi). Prazosin (0.1–0.2 mg/kg) almost completely reversed the increase in T_UP_ induced by D-amphetamine in both the low (*n* = 14) and high-amphetamine groups (*n* = 11). Injections of additional doses of D-amphetamine (up to 4.5 mg/kg) after prazosin slightly decreased T_UP_ in the low-amphetamine group (*p* < 0.01) and produced no further effects in the high-amphetamine group (*p* = 1). Prazosin completely reversed the effect of D-amphetamine on P_SO_ in both the low and high-amphetamine groups. In both groups, additional doses of D-amphetamine produced no further effects (low *p* = 0.405, high *p* = 1, Figures 3Ciii,v).

Figure 3D compares effects of prazosin with those of SCH23390 and raclopride. In both the low and high-amphetamine groups, prazosin was more effective than SCH23390 and raclopride in reversing the effect of D-amphetamine on T_UP_ (Figure 3Di). In the high, but not low-amphetamine group, prazosin was also more effective than SCH23390 and raclopride in reversing D-amphetamine’s effect on P_SO_ (Figure 3 Dii). There were no significant differences between SCH23390 and raclopride in their ability to reverse D-amphetamine’s effects.

### Effects of Dopamine Receptor Agonists and L-DOPA

#### Activation of Either D1 or D2-like Receptors Alone Was Ineffective in Increasing T_UP_

**FIGURE 4:**
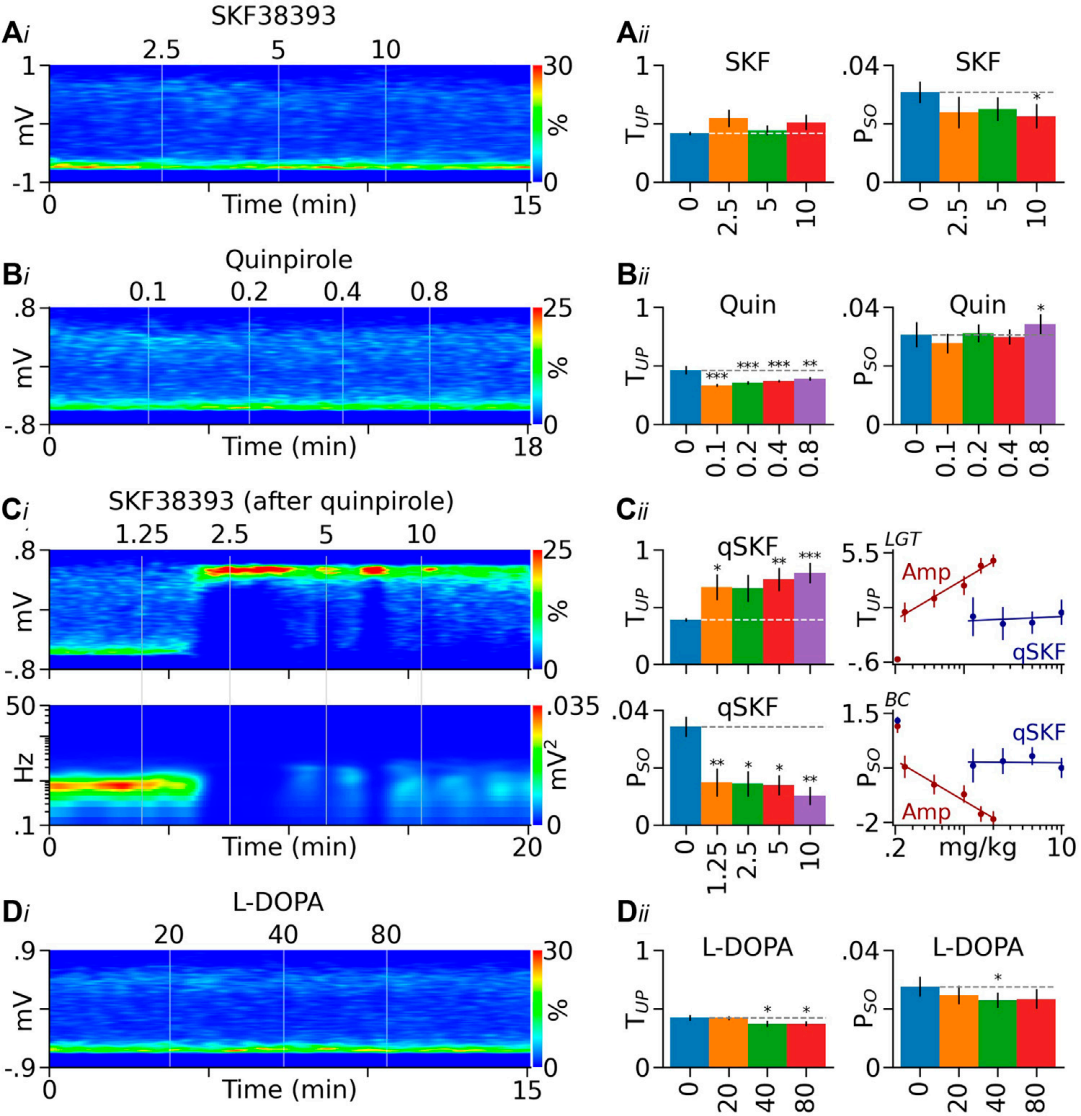
Effects of DA receptor agonists and L-DOPA on PFC Up state. **(Ai)** Heatmap from a typical rat showing the lack of a significant effect of the D1-like receptor agonist SKF38393 (SKF) on PFC LFP distribution. **(Aii)** Summary graphs showing that SKF38393 produced no significant effect on T_UP_ (left) and induced a small decrease in P_SO_ (right). **(Bi)** Heatmap from another rat showing that the D2-like receptor agonist quinpirole (Quin) slightly shifted LFPs toward the Down state. **(Bii)** Summary graphs showing that the decrease in T_UP_ induced by quinpirole was significant (left). Quinpirole also induced a small increase in P_SO_ (right). **(Ci)** Heatmap from a quinpirole-pretreated rat (0.8 mg/kg) showing that SKF38393 (qSKF) promoted the Up state (top) and suppressed the slow oscillation (bottom). **(Cii)** Summary graphs showing that in quinpirole-pretreated rats, SKF38393 significantly increased T_UP_ and decreased P_SO_ (left). Its dose-response curves are significantly right -shifted, however, relative to those of D-amphetamine (Amp, right). The *y*-axis values in dose-response curves are logit (LGT) or Box-Cox (BC) transformed. **(Di)** Heatmap from another rat showing that the DA/NE precursor L-DOPA slightly shifted PFC LFPs toward the Down state. **(Dii)** Summary bar graphs showing that the decrease in T_UP_ induced by L-DOPA was significant (left). The drug also produced a small decrease in P_SO_ (right). *p < 0.05, **p < 0.01, ***p < 0.001 vs baseline (blue bars).

SKF38393 (2.5–10 mg/kg, *n* = 11, Figure 4A) produced no significant effects on T_UP_ (F_3,31_ = 1.13, *p* = 0.352) and induced a small inhibition of P_SO_ at its highest dose (F_3,22_ = 3.945, *p* < 0.05). Quinpirole (0.1–0.8 mg/kg, *n* = 6, Figure 4B) slightly decreased T_UP_ (F_4,24_ = 25.52, *p* < 0.001) and induced a small increase in P_SO_ at its highest dose (F_4,24_ = 7.407, *p* < 0.001).

#### Co-activation of D1 and D2-like Receptor Partially Mimicked the Effect of D-Amphetamine

To test whether concurrent activation of D1 and D2-like receptors mimics the effects of D-amphetamine, we injected SKF38393 (1.25–10 mg/kg) in rats pretreated with quinpirole (0.8 mg/kg, *n* = 6, Figure 4C). SKF38393 significantly increased T_UP_ (F_4,24_ = 6.248, *p* < 0.001) and decreased P_SO_ (F_2,24_ = 6.142, *p* < 0.001). Compared to D-amphetamine, however, SKF38393’s effects were significantly reduced (T_UP_: intercept F_1,32_ = 9.914, *p* < 0.01, slope F_1,63_ = 14.05, *p* < 0.001; P_SO_: intercept F_1,27_ = 6.019, *p* < 0.05, slope F_1,63_ = 17.57, *p* < 0.001, Figure 4C).

#### L-DOPA Did Not Mimic the Effect of D-Amphetamine

In a separate group of rats (*n* = 7), we administered the DA/NE precursor L-DOPA (20–80 mg/kg). Unlike D-amphetamine, L-DOPA decreased T_UP_ (F_3,21_ = 7.557, *p* = 0.001), instead of increasing it. L-DOPA also produced a small decrease in P_SO_ (F_4,24_ = 3.985, *p* < 0.05, Figure 4D).

## Discussion

We have previously shown that D-amphetamine promotes PFC Up state and the effect is blocked by antagonists at α1 adrenergic receptors (Shen and Shi, 2021). In this study, we confirm these findings and further show that DA receptors also play a role in the effect of D-amphetamine. Furthermore, we show that D-amphetamine’s effect is state-dependent, decreasing with decreasing baseline T_UP_. These results provide new insights into the multifaceted action of D-amphetamine and may have important clinical implications.

### Role of Norepinephrine in D-Amphetamine’s Effect

The transition from Up/Down oscillations to a persistent Up state has been suggested to play a critical role in arousal and attention (Harris and Thiele, 2011; Vazey and Aston-Jones, 2014; Mcginley et al., 2015; Poulet and Crochet, 2019). Given that, the Up-state promoting effect of D-amphetamine is consistent with its efficacy in treating attention-deficit/hyperactivity disorder (ADHD) and narcolepsy (Heal et al., 2013) and with its ability to accelerate emergence from general anesthesia (Kenny et al., 2015). In line with a role of NE in D-amphetamine’s effect, activation of NE neurons in the locus coeruleus promotes cortical Up state (Steriade et al., 1993; Dringenberg and Vanderwolf, 1997; Durán et al., 2021), induces an immediate transition from sleep to wakefulness (Carter et al., 2010), and accelerates emergence from anesthesia (Vazey and Aston-Jones, 2014). These effects, like D-amphetamine’s Up-state promoting effect, are blocked by the α1 receptor antagonist prazosin. Local infusion of NE in the PFC also promotes the Up state as indicated by a transition in EEG from large-amplitude, low-frequency waves to low-amplitude, high-frequency oscillations (Pal et al., 2018). Local application of NE receptor antagonists, on the other hand, blocks the transition from UP/Down oscillations to a persistent Up-state during emergence from general anesthesia (Constantinople and Bruno, 2011). In this study, we provided evidence that the effect of D-amphetamine involves activation of not only α1 adrenergic receptors, but also D1 and D2-like DA receptors.

### Role of Dopamine Receptors in D-Amphetamine’s Effect

A role for DA receptors in D-amphetamine’s effect was first suggested by our observation that the effect was significantly attenuated in rats pretreated with the D1-like receptor antagonist SCH23390, the D2-like receptor antagonist raclopride, or both. The two DA antagonists also partially reversed D-amphetamine’s effect when given after D-amphetamine. A role of DA in D-amphetamine’s effect is further supported by our finding that co-activation of D1 and D2-like receptors partially mimics the effect of D-amphetamine. The selective DA transporter blocker GBR12909 has also been shown to partially mimic the effect of D-amphetamine (Shen and Shi, 2021). In line with our results, D-amphetamine increases the release of both NE and DA (Kuczenski and Segal, 1997; Rothman et al., 2001) and has been shown to accelerate emergence from anesthesia via DA receptors (Taylor et al., 2013; Kenny et al., 2015). Furthermore, activation of DA neurons in the ventral tegmental area is known to induce an Up state-like depolarization in PFC pyramidal neurons (Lewis and O’donnell, 2000; Onn and Wang, 2005; Iwashita, 2014; Gretenkord et al., 2020), accelerate emergence from anesthesia (Solt et al., 2014; Taylor et al., 2016), and promote wakefulness (Eban-Rothschild et al., 2016; Oishi et al., 2017a; Sun et al., 2017). Additional evidence suggests that DA produces these effects, at least in part, *via* D1 and D2-like receptors in the nucleus accumbens (Eban-Rothschild et al., 2016; Oishi et al., 2017b; Luo et al., 2018; Zhang et al., 2021).

### Dopamine-Norepinephrine Interaction in D-Amphetamine’s Effect

It is important to point out that DA receptor antagonists only attenuated the effect of D-amphetamine, whereas the α1 receptor antagonist prazosin completely blocked it. This observation raises the possibility that the DA-mediated portion of D-amphetamine’s effect requires co-activation of α1 receptors. A potential site through which DA and NE interact to promote the Up state is the glutamate NMDA receptor. Evidence suggests that activation of NMDA receptors is essential to the generation of UP states (see review by Neske, 2016). Consistent with this suggestion, we have shown that the selective NMDA receptor antagonist MK801 as well as ketamine inhibits PFC Up state (Shen et al., 2018). We and others also show that D1 receptor activation enhances NMDA, but not AMPA, receptor-mediated currents in PFC pyramidal neurons (Zheng et al., 1999; Tseng and O’donnell, 2004). Since NMDA-receptor channels are blocked by Mg^2+^ at the resting membrane potential and open only when the cell is sufficiently depolarized by a large glutamate release, and since NE increases glutamate release (Marek and Aghajanian, 1999; Luo et al., 2014; Luo et al., 2015), it is possible that DA promotes the Up state only when glutamate release is sufficiently increased by NE to evoke NMDA receptor currents. Thus, by blocking α1 receptors and decreasing glutamate release, prazosin may indirectly block the DA-induced enhancement of NMDA receptor currents.

Studies by Darracq et al. suggest another possibility. In their studies, prazosin, given systemically or locally in the PFC, prevents D-amphetamine from inducing locomotor activation and increasing “functional DA release” in the nucleus accumbens (Darracq et al., 1998). Selective NE depletion in the PFC also inhibits D-amphetamine’s ability to induce conditioned place preference and DA release in the nucleus accumbens (Ventura et al., 2003; see also review by; Dela Peña et al., 2015). Thus, D-amphetamine may act through PFC α1 receptors to increase DA release in the nucleus accumbens which then activates D1 and D2-like receptors to contribute to the Up-state promoting effect of D-amphetamine. Consequently, by blocking PFC α1 receptors and inhibiting D-amphetamine-induced DA release in the nucleus accumbens, prazosin also blocks the DA-mediated portion of D-amphetamine’s effect on PFC Up state. Consistent with this possibility, DA in the nucleus accumbens has been suggested to promote wakefulness and accelerate emergence from anesthesia (Eban-Rothschild et al., 2016; Oishi et al., 2017b; Luo et al., 2018; Zhang et al., 2021).

### Dopamine’s Role Varies Depending on the Dose of D-Amphetamine

Also different from prazosin which was equally effective in blocking the effects induced by low and high doses of D-amphetamine, SCH23390 and raclopride were found to be more effective in reversing the effects induced by low doses of D-amphetamine than those induced by high doses. However, raclopride increased T_UP_ when given after SCH23390. Conflicting results were also obtained when the two DA antagonists were given before D-amphetamine. Thus, SCH23390 pretreatment induced only a small change in the slope of D-amphetamine’s dose-response curve. Raclopride pretreatment, on the other hand, preferentially reduced the effect induced by high doses of D-amphetamine. In rats pretreated with both SCH23390 and raclopride, D-amphetamine exhibited an inverted U-shaped dose-response curve on T_UP_, suggesting an attenuation of effects induced by low and high, but not intermediate, doses of D-amphetamine.

Part of the inconsistencies may be attributed to the fact that all drugs in this study were administered systemically. They may thus act through multiple brain areas to produce different and possibly opposing influences on D-amphetamine’s effect. For example, blockade of DA receptors in the PFC and nucleus accumbens may attenuate the effect of D-amphetamine, but blockade of D2-like receptors on DA neurons (DA autoreceptor) would produce the opposite effect. We have shown that raclopride, by blocking DA autoreceptors, not only prevents D-amphetamine from inhibiting DA neurons, but also enables the stimulant to excite DA neurons via α1 receptors (Shi et al., 2000a). Our evidence suggests that D1-like receptors are also involved in feedback regulation of DA neurons (Shi et al., 1997) and D-amphetamine inhibits DA neurons via both D1 and D2-like receptors (Shi et al., 2000b). The observation that a DA antagonist differentially alters D-amphetamine’s effect depending on whether it is given before and after D-amphetamine further raises the possibility that different processes are involved in the initiation and maintenance of D-amphetamine’s effect. Thus, more studies will be needed to test whether DA’s role differs depending on not only the dose of D-amphetamine, but also the stage of D-amphetamine’s effect.

### Effects of Dopamine Receptor Agonists and L-DOPA

While co-activation of D1 and D2-like receptors partially mimicked the effect of D-amphetamine, we found that activation of either D1 or D2-like receptors alone was ineffective, indicating an interdependence between D1 and D2-like receptors in regulating cortical Up state. We have previously shown that the expression of the D1 receptor-mediated feedback inhibition of DA neurons also requires co-activation of D2-like receptors (Shi et al., 1997; Shi et al., 2000b). Unexpectedly, the DA precursor L-DOPA, which increases DA release and would indirectly activate both D1 and D2-like receptors, also failed to mimic the effect of D-amphetamine. L-DOPA is also a precursor for NE and increases NE release (Dayan and Finberg, 2003). The exact reason for the failure of L-DOPA to mimic D-amphetamine’s effect is unknown, but it may be related to the pattern of DA release induced by L-DOPA. We have shown that psychostimulants, including D-amphetamine and cocaine, induce slow oscillations in the firing activity of DA neurons (Shi et al., 2004; Zhou et al., 2006), and the oscillations are temporally correlated to the Up/Down oscillations seen in PFC neurons (Gao et al., 2007). We speculate that oscillatory firing of DA neurons leads to oscillatory DA release in the nucleus accumbens and that DA promotes Up states only when its release is temporally correlated with the oscillatory activity of PFC neurons (see review by Dela Peña et al., 2015). Unlike psychostimulants, L-DOPA induces pacemaker-like firing in DA neurons (Shi et al., 2004; Zhou et al., 2006; Xu et al., 2011), predicting that L-DOPA induces a tonic DA release that is temporally uncorrelated with PFC activity. While further experiments are needed to test this and other possibilities, our results are consistent with the fact that L-DOPA is not a psychostimulant and is ineffective in treating ADHD (Overtoom et al., 2003; England et al., 2011). The inability of L-DOPA to promote cortical Up state further predicts that the drug is ineffective in treating excess daytime sleepiness including narcolepsy and in accelerating emergence from general anesthesia.

### Dependence of D-Amphetamine’s Effect on Brain States

We found in this study that the brain state, measured by T_UP_ before D-amphetamine injection, varied between animals. This variation is likely to be caused by different levels of anesthesia since deepening of anesthesia has been shown to prolong Down state duration, thus decreasing T_UP_ (Dasilva et al., 2021). We also found that D-amphetamine was less effective in promoting Up states in rats with low baseline T_UP_ than those with high baseline T_UP_. One possible explanation for this observation is that D-amphetamine promotes Up states by enhancing synaptic inputs responsible for the generation of Up states and these synaptic inputs are suppressed when baseline T_UP_ is low. Supporting this possibility, cortical Up state is known to depend on glutamate inputs, and both NE and DA modulate glutamate transmission in the PFC (see *DA-NE Interaction in D-Amphetamine’s Effect* above).

The state-dependence of D-amphetamine’s effect may have important clinical implications. Studies suggest that psychostimulants, including D-amphetamine and methylphenidate, accelerate emergence from general anesthesia (Solt et al., 2011; Chemali et al., 2012; Kenny et al., 2015; Moody et al., 2020). The state-dependence of D-amphetamine’s effect predicts that these agents are less effective in reversing anesthesia when the level of anesthesia is relatively deep. As discussed in the Introduction, Up/Down oscillations also occur locally during wakefulness, increase with increasing sleep pressure, and have profound influence on sensory processing and behavioral performance. The state-dependence of D-amphetamine’s effect predicts that D-amphetamine affects brain areas differentially based on the local levels of T_UP_. Specifically, D-amphetamine may be less effective in regions where the Down state is dominant. Future studies are needed to test this possibility and to determine whether the differential effects of D-amphetamine on various cortical regions correlate with its effects on sensory, motor, and cognitive functions.

### Limitations

There are several caveats in the present study. First, all drugs were given systemically. For that, the location of the DA receptors responsible for the effect of D-amphetamine remains to be determined. As suggested above, D-amphetamine may act through multiple areas to product its effects, including the PFC, nucleus accumbens, and ventral tegmental area. More detailed studies will be needed to determine how DA in each of these areas contributes to D-amphetamine’s effect. Second, all experiments were done in male rats. Significant sex differences have been observed in D-amphetamine’s reinforcing and psychotomimetic effects (e.g., Vansickel et al., 2010; Gogos et al., 2017), raising the possibility that D-amphetamine’s effect on cortical Up state also exhibits a sex difference. Third, the D1-like receptor agonist used in this study SKF38393 is a partial agonist. While it increased T_UP_ in quinpirole-pretreated rats, the effect was weaker than that observed with D-amphetamine. SKF38393 also showed a relatively flat dose-response curve. Experiments will be needed to determine whether those properties of SKF38393 are shared by a full D1 agonist and unrelated to its partial agonist properties. Fourth, the D2-like receptor antagonist used in this study raclopride is believed to block D_2_ and D_3_, but not D_4_ receptors. Thus, the role of D_4_ receptors in D-amphetamine’s effect remains to be investigated. Finally, this study showed that SCH23390 and raclopride were more effective in reversing the effect of D-amphetamine in the low-amphetamine group than in the high-amphetamine group. Since high doses of D-amphetamine required more time to inject (see Figure 3), it remains possible that in addition to the difference in dose, the difference in injection time also contributes to the difference in SCH23390 and raclopride’s ability to reverse D-amphetamine’s effect between the high and low-amphetamine groups. More studies will be needed to rule out this possibility.

## Conclusion

This study shows that the Up-state promoting effect of D-amphetamine involves activation of not only α1 NE receptors, but also D1 and D2-like DA receptors. Our results are consistent with the fact that D-amphetamine increases the release of both NE and DA and are in line with D-amphetamine’s efficacy in treating ADHD and narcolepsy and in reversing general anesthesia. Our results are also in agreement with studies showing that DA promotes wakefulness and mediates, at least in part, the reversal of anesthesia induced by D-amphetamine. Our results also suggest an interdependence between D1 and D2-like receptors in the effect of D-amphetamine. Unexpectedly, the DA/NE precursor L-DOPA failed to mimic the effect of D-amphetamine. While more studies are needed to understand the difference between L-DOPA and D-amphetamine, our finding may provide an explanation for why L-DOPA lacks significant psychostimulant properties and is ineffective in treating ADHD.

## Data Availability

The original contributions presented in the study are included in the article/Supplementary Material, further inquiries can be directed to the corresponding author.
